# Intracellular Renin Inhibits Mitochondrial Permeability Transition Pore via Activated Mitochondrial Extracellular Signal-Regulated Kinase (ERK) 1/2 during Ischemia in Diabetic Hearts

**DOI:** 10.3390/ijms19010055

**Published:** 2017-12-25

**Authors:** Terumori Satoh, Masao Saotome, Hideki Katoh, Daishi Nonaka, Prottoy Hasan, Hideharu Hayashi, Yuichiro Maekawa

**Affiliations:** Internal Medicine III, Hamamatsu University School of Medicine, Hamamatsu 431-3192, Japan; terumori@hama-med.ac.jp (T.S.); hkatoh56@gmail.com (H.K.); daishinonaka@gmail.com (D.N.); dr.prottoyhasan@gmail.com (P.H.); hideharuhayashi@gmail.com (H.H.); ymaekawa@hama-med.ac.jp (Y.M.)

**Keywords:** mitochondrial ERK1/2, diabetes mellitus, ischemia, intracellular renin, mitochondrial permeability transition pore

## Abstract

Although beneficial effects of non-secreting intracellular renin (ns-renin) against ischemia have been reported, the precise mechanism remains unclear. In this study, we investigated the roles of ns-renin and mitochondrial extracellular signal-related kinase (ERK) 1/2 on mitochondrial permeability transition pore (mPTP) opening during ischemia in diabetes mellitus (DM) hearts. When isolated hearts from Wistar rats (non-DM hearts) and Goto-Kakizaki rats (DM hearts) were subjected to ischemia for 70 min by left anterior descending coronary artery ligation, DM hearts exhibited higher left ventricular (LV) developed pressure and lower LV end-diastolic pressure than non-DM hearts, suggesting ischemic resistance. In addition, DM hearts showed increased intracellular renin (int-renin, including secreting and non-secreting renin) in the ischemic area, and a direct renin inhibitor (DRI; aliskiren) attenuated ischemic resistance in DM hearts. ERK1/2 was significantly phosphorylated after ischemia in both whole cell and mitochondrial fractions in DM hearts. In isolated mitochondria from DM hearts, rat recombinant renin (r-renin) significantly phosphorylated mitochondrial ERK1/2, and hyperpolarized mitochondrial membrane potential (ΔΨ_m_) in a U0126 (an inhibitor of mitogen-activated protein kinases/ERK kinases)-sensitive manner. R-renin also attenuated atractyloside (Atr, an mPTP opener)-induced ΔΨ_m_ depolarization and Atr-induced mitochondrial swelling in an U0126-sensitive manner in isolated mitochondria from DM hearts. Furthermore, U0126 attenuated ischemic resistance in DM hearts, whereas it did not alter the hemodynamics in non-DM hearts. Our results suggest that the increased int-renin during ischemia may inhibit mPTP opening through activation of mitochondrial ERK1/2, which may be involved in ischemic resistance in DM hearts.

## 1. Introduction

It is widely accepted that the Renin-Angiotensin System (RAS) plays a central role in the pathogenesis of heart diseases, such as hypertension, cardiac hypertrophy, and heart failure [1]. Renin is a secretory glycoprotein and generates angiotensin I from angiotensinogen. Angiotensin I is further cleaved to angiotensin II by angiotensin-converting enzyme, which is the effector peptide of RAS, and promotes hypertrophy, apoptosis, necrosis, fibrosis, and remodeling of myocardium. Nevertheless, intensive recent investigations revealed that local RAS plays a significant pathophysiological role in heart disease. In the heart, emerging evidence has revealed that renin transcript codes for exon (1A-9) renin (non-secreting intracellular renin; ns-renin), which is derived from alternative renin transcript, is increased by myocardial infarction [2]. We and others reported that ns-renin increases in the myocardium after myocardial ischemia [2,3,4] and is involved in cardiac protection during ischemia [4,5,6]. However, the precise mechanism by which increased ns-renin protects the heart from ischemia remains unclear.

It is widely accepted that the mitochondrial permeability transition pore (mPTP) is a major cause of ischemia-reperfusion (I/R) injury, and inhibiting mPTP affords the cardiac protection against I/R injury [7]. Because the ns-renin mainly accumulates in mitochondria [8], it would be possible that mitochondrial ns-renin may inhibit mPTP through modulation of mitochondrial function during ischemia. In addition, independently from angiotensin II production, renin can activate intracellular signaling through binding (pro) renin receptor ((P)RR). Although the binding ns-renin with (P)RR remains elusive, ns-renin may trigger downstream signal molecules of (P)RR, some of which are related with pro-survival kinase-signaling cascades in ischemic preconditioning [9] and inhibitors of mPTP opening [10].

Hence, in the present study, we investigated first the roles of intracellular renin (int-renin) on activation of signal molecules in ischemic resistance of diabetes mellitus (DM) hearts and second the contribution of mPTP to int-renin-mediated ischemic resistance in DM hearts.

## 2. Results

### 2.1. Intracellular Renin is Relevant for Ischemic Resistance in DM Hearts

**Figure 1:**
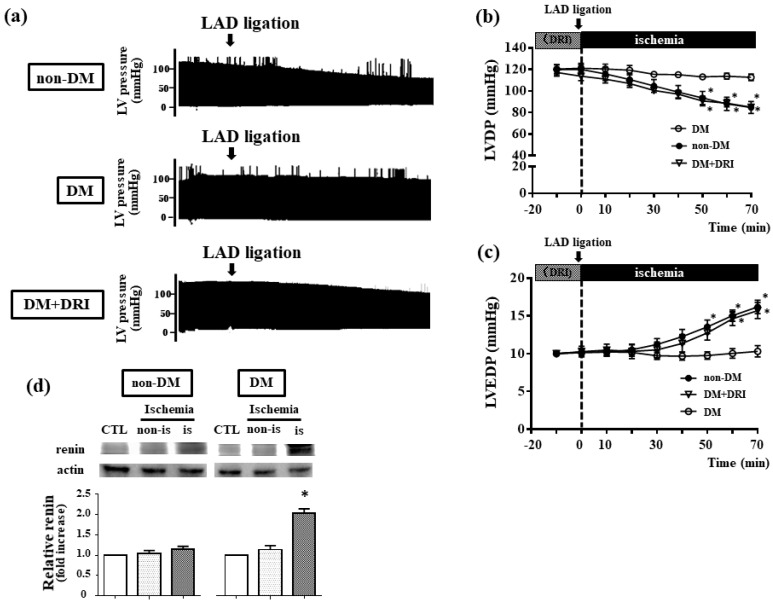
DM hearts exhibit ischemic resistance through ERK1/2 activation. (**a**) Representative recordings of left ventricular (LV) pressure in non-DM and DM hearts. Langendorff-perfused hearts were subjected to 70 min ischemia by left anterior descending artery (LAD) ligation. A direct renin inhibitor (DRI; aliskiren) was added 20 min before coronary ligation; (**b**,**c**) Time courses of changes in left ventricular developed pressure (b: LVDP) and left ventricular end-diastolic pressure (c: LVEDP) during ischemia in non-DM (●, *n* = 6), DM (○, *n* = 6), and DM + DRI (∇, *n* = 4). Values are the means ± standard error of the mean SEM. * *p* < 0.05 vs. non-DM hearts by two-way analysis of variance (ANOVA); (**d**) Western blot analysis of renin in both non-DM and DM hearts. The samples were obtained from the non-ischemic hearts (CTL), non-ischemic area (non-is), and ischemic area (is) from ischemic hearts. Data are presented as fold-increase of renin/actin ratio from CTL, and values are the means ± SEM from 3 isolated experiments. * *p* < 0.05 vs non-ischemic control (CTL) of DM heats by two-way ANOVA.

To evaluate roles of ns-renin in ischemic resistance of DM hearts, 10-week-old Goto-Kakizaki (DM) rats were used. In Table 1, DM rats exhibited higher plasma levels of glucose, glycoalbumin, and glycohemoglobin and lower plasma levels of insulin in laboratory examination. In addition, DM rats showed higher heart weight and heart weight/bodyweight ratio than those of non-DM hearts, suggesting cardiac hypertrophy due to diabetic cardiomyopathy [11]. In the hemodynamic analysis, baseline data did not differ between DM and non-DM hearts (Supplementary Materials Figure S1). When isolated hearts from DM rats were subjected to regional ischemia by LAD coronary artery ligation (Figure 1a), LV developed pressure (LVDP) was higher (113 ± 3 mmHg, *p* < 0.05 vs. 90 ± 7 mmHg of non-DM hearts, Figure 1b), and LV end-diastolic pressure (LVEDP) was lower than those of non-DM hearts (10.3 ± 0.7 mmHg, *p* < 0.05 vs. 15.9 ± 0.8 mmHg of non-DM hearts, Figure 1c). DM hearts exhibited increased int-renin, especially at the ischemic area (Figure 1d), and application of DRI attenuated ischemic resistance in DM hearts (LVDP; 85 ± 3 mmHg, *p* < 0.05 vs. 113 ± 3 mmHg of DM hearts, LVEDP; 15.7 ± 1.1 mmHg, *p* < 0.05 vs. 10.3 ± 0.7 mmHg of DM hearts), compatible with our previous results [4]. Thus, our results suggested that int-renin may be involved in ischemic resistance of DM hearts.

### 2.2. Roles of Mitochondrial ERK1/2 in Intracellular Renin-Mediated Ischemic Resistance

**Figure 2:**
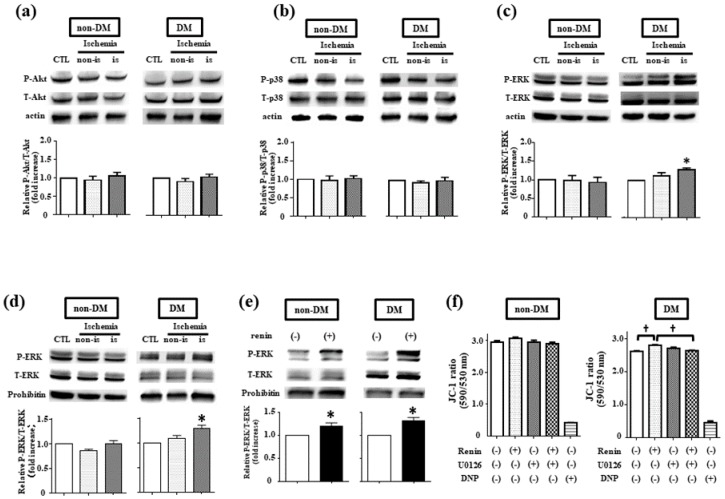
Pro-survival signals activation by rat recombinant renin (r-renin) and ischemia. Western blot analysis of intracellular signaling molecules, including Akt (**a**), p38 (**b**), and extracellular signal-regulated kinase 1/2 (ERK1/2): (**c**) in both non-DM and DM hearts. Hearts were subjected to ischemia for 70 min, and phosphorylation levels of intracellular signaling molecules (P-Akt, P-p38, and P-ERK) were assessed among the control, non-ischemic area (non-is), and ischemic area (is). Data represent the fold-increase of relative intensities in phosphorylated/total intracellular signaling molecules (T-Akt, T-p38, and T-ERK), and values are the means ± SEM from four independent experiments. * *p* < 0.05 vs. non-treated control (one-way ANOVA); (**d**) Representative Western blot analysis of total mitochondrial ERK1/2 and phosphorylated mitochondrial ERK1/2 both in non-DM and DM hearts. Hearts were subjected to ischemia for 70 min, and then mitochondria were isolated as described in the Materials and Methods. The phosphorylation levels of mitochondrial ERK1/2 were assessed in the non-ischemic area (non-is) and ischemic area (is). Non-ischemic heart was used as a control (CTL). Data are presented as fold-increase of phosphorylated/total mitochondrial ERK1/2 from CTL, and values are the means ± SEM from seven isolated experiments; (**e**) Western blot analysis of mitochondrial ERK1/2 phosphorylation. Isolated mitochondria from non-DM and DM hearts were exposed to rat recombinant renin (r-renin, 100 ng/mL) for 30 min, and phosphorylation levels of ERK1/2 were evaluated. Bars indicate relative intensities of phosphorylated/total ERK1/2, and values are the means ± SEM from five experiments. * *p* < 0.05 vs. control (one-way ANOVA); (**f**) Summarized data of 5,5′,6,6′-tetrachloro-1,1′,3,3′-tetraethylbenzimidazol-carbocyanine iodide (JC-1) in isolated mitochondria from non-DM and DM hearts. Isolated mitochondria were exposed to r-renin (100 ng/mL) for 30 min in the presence or absence of U0126 (1 µM). As a reference, an uncoupler of DNP (100 µmol/L) was applied to mitochondria both from DM and non-DM hearts. Values are the means ± SEM from three different experiments. * *p* < 0.05, ^†^ *p* < 0.01 vs. non-treated control (one-way ANOVA).

Because binding renin or prorenin with (P)RR can activate intracellular signaling such as mitogen-activated protein kinase (MAPK) [1,12], we next investigated alterations in intracellular signaling molecules including Akt, p38, and ERK1/2, which are involved in downstream signaling of (P)RR. Activation of these signaling molecules was evaluated using tissue from ischemic hearts (whole cell fraction) by LAD coronary artery ligation. Compared with the non-treated control, phosphorylation of ERK1/2 was activated in DM hearts (Figure 2c), whereas Akt and p38 were not (Figure 2a,b).

Because ns-renin mainly accumulates in mitochondria [5], we examined mitochondrial ERK1/2 in the roles of ischemic resistance using isolated mitochondria from DM hearts. In Figure 2d, ischemic stimulation significantly activated mitochondrial ERK1/2 phosphorylation in DM hearts, whereas did not in non-DM hearts. The causal relationship between int-renin and mitochondrial ERK1/2 was further confirmed using isolated mitochondria and recombinant rat renin (r-renin). When isolated mitochondria were exposed to r-renin, mitochondrial ERK1/2 was significantly phosphorylated in DM hearts and also in non-DM hearts (Figure 2e).

We further examined functional alterations in the mitochondria after r-renin exposure (Figure 2f). Mitochondria from DM hearts exhibited depolarized ΔΨ_m_ compared with those of non-DM hearts at the baseline (JC-1 ratio; 2.96 ± 0.04 of non-DM, vs. 2.63 ± 0.01 of DM). Although r-renin did not alter ΔΨ_m_ in non-DM hearts, it hyperpolarized ΔΨ_m_ in DM hearts (JC-1 ratio; 2.81 ± 0.01 of renin, *p* < 0.05 vs. 2.63 ± 0.01 of control). When isolated mitochondria were pretreated with U0126, the ΔΨ_m_ hyperpolarization by r-renin was attenuated in DM hearts (2.64 ± 0.04 of renin + U0126, *p* < 0.05 vs. 2.81 ± 0.01 of renin). These results suggest that int-renin may hyperpolarize ΔΨ_m_ through mitochondrial ERK1/2 activation in DM hearts.

### 2.3. R-Renin Inhibited Atractyloside-Induced mPTP Opening in DM Hearts

**Figure 3:**
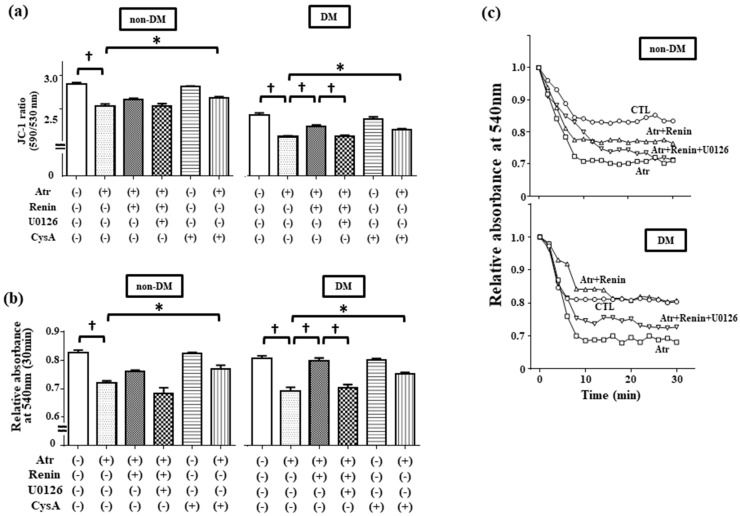
Rat recombinant renin (r-renin) inhibited atractyloside (Atr)-induced mitochondrial permeability transition pore (mPTP) opening by activating mitochondrial ERK1/2 in DM hearts. (**a**) Summarized data of JC-1 ratio in isolated mitochondria from non-DM and DM hearts. Isolated mitochondria were exposed to Atr (50 µM) to open mPTP in the presence or absence of r-renin (100 ng/mL). Some mitochondria were incubated with U0126 (1 µM) or cyclosporine A (CysA, 1 µM) and then the JC-1 ratio in each condition was monitored. Values are the means ± SEM of 24 different experiments. * *p* < 0.05, ^†^ *p* < 0.01 vs. non-treated control (one-way ANOVA); (**b**) Summarized data of relative light scattering at 30 min. Isolated mitochondria were exposed to Atr (50 µM) in the presence or absence of r-renin (100 ng/mL). Some mitochondria were incubated with U0126 (1 µM) or CysA (1 µM) and light absorbance in each condition was monitored. Data are relative light absorbance at 540 nm from time point 0 (without treatment). Values are the means ± SEM from five different experiments. * *p* < 0.05, ^†^ *p* < 0.01 (two-way ANOVA); (**c**) Representative time course of changes in mitochondrial swelling in isolated mitochondria from non-DM and DM hearts. Isolated mitochondria were exposed to Atr in the absence (Atr: □) or presence of r-renin (Atr + Renin: △). Some mitochondria were pretreated with U0126 (Atr + Renin + U0126: ▽). The non-treated control was referred to as the control (○). Data are relative light absorbance at 540 nm from time point 0 (without treatment).

Because mPTP opening plays crucial roles in ischemic injury, we next investigated the effects of r-renin on mPTP. In Figure 3a, when isolated mitochondria were exposed to Atr, ΔΨ_m_ was depolarized in a CysA-sensitive manner both in non-DM and DM hearts, suggesting that Atr depolarized ΔΨ_m_ through mPTP opening. R-renin attenuated Atr-induced ΔΨ_m_ depolarization in DM hearts (2.25 ± 0.02 of Atr + Renin, *p* < 0.05 vs. 2.09 ± 0.01 of Atr), whereas did not significantly alter in non-DM hearts. In addition, U0126 attenuated this effect of r-renin in DM hearts (2.10 ± 0.01 of Atr + Renin + U0126, *p* < 0.05 vs. 2.25 ± 0.02 of Atr + Renin, Figure 3a). The effect of r-renin on Atr-induced mPTP opening was further evaluated in a mitochondrial swelling assay. As shown in Figure 3b,c, Atr induced mitochondrial swelling in a CysA-sensitive manner both in non-DM and DM hearts, suggesting that Atr opened mPTP. R-renin attenuated Atr-induced mitochondrial swelling in DM hearts (the ratio to baseline, 0.80 ± 0.01 of Atr + Renin, *p* < 0.05 vs. 0.69 ± 0.01 of Atr), whereas it did not significantly alter in non-DM hearts. U0126 attenuated this effect of r-renin in DM hearts (the ratio to baseline, 0.70 ± 0.01 of Atr + Renin + U0126, *p* < 0.05 vs. 0.80 ± 0.01 of Atr + Renin). Thus, our results suggested that int-renin may inhibit mPTP opening through mitochondrial ERK1/2 activation, and the protective effects of int-renin are specific to DM hearts.

### 2.4. Inhibition of ERK1/2 Abolished Ischemic Resistance in DM Hearts

**Figure 4:**
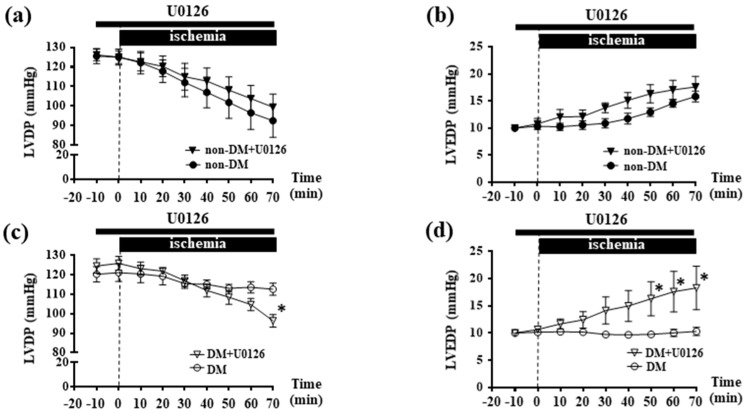

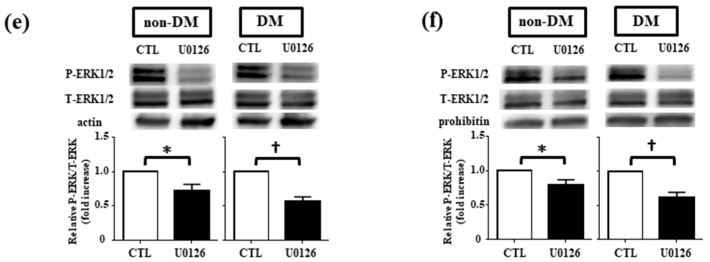
Effects of U0126 on ischemic resistance in DM hearts. Time courses of changes in LVDP (**a**) and LVEDP (**b**) during ischemia in non-DM hearts in the absence (●: *n* = 6) or presence of U0126 (▼: *n* = 7). Values are means ± SEM. Time courses of changes in LVDP (**c**) and LVEDP (**d**) during ischemia in DM hearts in the absence (○: *n* = 6) or presence of U0126 (▽: *n* = 4). Values are the means ± SEM. * *p* < 0.05 vs. DM by two-way ANOVA. Effect of U0126 on whole cell ERK1/2 (**e**) and mitochondrial ERK1/2 (**f**). Representative Western blot analysis of ERK1/2 in both non-DM and DM hearts. Langendorff-perfused hearts were treated with U0126 for 70 min, and the phosphorylation levels of ERK1/2 were assessed. Data represent the fold-increase of phosphorylated/total ERK1/2 from non-treated CTL, and values are the means ± SEM of four independent experiments. * *p* < 0.05, ^†^ *p* < 0.01 vs. CTL (one-way ANOVA).

We finally evaluated the contribution of mitochondrial ERK1/2 to ischemic resistance in DM hearts using U0126. When isolated hearts were perfused with U0126, where cytosolic and mitochondrial ERK1/2 were significantly suppressed both in non-DM and DM hearts (Figure 4e,f), U0126 abolished ischemic resistance of DM hearts; i.e., U0126 decreased LVDP (DM hearts with U0126; 96.4 ± 3.2 mmHg, *p* < 0.05 vs. 112.7 ± 3.1 mmHg of DM hearts, Figure 4c) and increased LVEDP during ischemia in DM hearts (DM hearts with U0126; 18.3 ± 4 mmHg *p* < 0.05 vs. 10.3 ± 0.7 mmHg of DM, Figure 4d), whereas did not alter in non-DM hearts (Figure 4a,b). These results suggest that ischemic resistance of DM hearts may be mediated through cytosolic and/or mitochondrial ERK1/2 activation.

## 3. Discussion

We studied the roles of intracellular signaling in ischemic resistance in DM hearts and the contribution of int-renin and subsequent mitochondrial signaling on mPTP inhibition during ischemia. The main findings of these experiments were as follows: (1) int-renin was involved in ischemic resistance in DM hearts, (2) DM hearts exhibited activated ERK1/2 during ischemia both in the whole cell and mitochondrial fractions, (3) r-renin activated mitochondrial ERK1/2, hyperpolarized ΔΨ_m_, and inhibited atractyloside-induced mPTP opening in DM hearts, and (4) pharmacological inhibition of ERK1/2 by U0126 attenuated the ischemic resistance in DM hearts.

### 3.1. Intracellular Renin Contributes to Ischemic Resistance in DM Heart

Although it is well known that DM is one of most critical exacerbation factors for ischemic heart disease, several DM animals have exhibited cardiac ischemic resistance [13]. Emerging evidence has revealed that the local RAS plays critical roles in the pathogenesis of cardiovascular disease [14], and ns-renin was reported to increase in the myocardium after myocardial ischemia [2,3,4]. Although the precise mechanism remains unclear, ns-renin is considered to supply cardiac protection during ischemia. In this study, DM hearts showed the ischemic resistance in the hemodynamic parameters (Figure 1a–c) and increased int-renin expression particularly in the ischemic area (Figure 1d). In addition, DRI inhibited the ischemic resistance in DM hearts (Figure 1a–c). Although we can exclude the influence of circulating RAS (because we used the isolated hearts with Langendorff-perfusion and application of angiotensin receptor blocker valsartan did not alter the ischemic resistance in DM hearts [4]), we cannot deny the possibility that secreting renin (instead of ns-renin) would contribute to the ischemic resistance in DM hearts via autocrine and/or paracrine signaling. This is because our results in DM hearts cannot identify which type of int-renin (non-secreting and/or secreting) increased during ischemia. However, ns-renin seems to be preferentially involved in ischemic resistance of DM hearts, because cardiac myocytes secretes less renin through exocytosis [1]. To better clarify the role of int-renin on ischemic resistance in DM hearts, we have assessed the int-renin mediated ischemic resistance using H9c2 cardiac myocytes. When the high glucose-treated H9c2 myocytes, which showed increased int-renin expression at baseline, were exposed to hypoxia and subsequent reoxygenation (H/R), cells exhibited further increased int-renin and suppressed the caspase-3 activity compared to normal-glucose-treated cells (Supplementary Materials Figure S3). In addition, suppressing renin by siRNA attenuated this high-glucose-mediated cell protection against H/R (Supplementary Materials Figure S4). From these results we consider that increased int-renin by hyperglycemia may afford the cell protection from H/R. Compatible with our results, Wanka et al. reported that overexpression of ns-renin protected H9c2 myoblasts from necrosis [5], and Langendorff-perfused hearts obtained from ns-renin overexpressing transgenic rats exhibited diminished ischemia-induced damage [6].

### 3.2. Mitochondrial ERK1/2 Activation is Involved in Ischemic Resistance in DM Hearts

In this study, we showed that ischemic stimulation activated not only cellular ERK1/2 (Figure 2c) but also mitochondrial ERK1/2 (Figure 2d) in DM hearts, whereas neither was significant in non-DM hearts. In addition, an inhibitor of mitogen-activated protein kinases/ERK kinases (U0126) attenuated the ischemic resistance in DM hearts (Figure 4). These results suggest that ERK1/2 activation is relevant in the mechanism of ischemic resistance in DM hearts. Our results cannot clearly identify whether activated ERK1/2 was translocated from cytosolic space or mitochondrial ERK1/2 was activated on the surface of mitochondria by int-renin accumulation during ischemia. This is because we evaluated the involvement of ERK1/2 in ischemic resistance of DM hearts using U0126, where both cellular and mitochondrial levels of ERK1/2 were significantly suppressed (Figure 4e,f). Further examination will be required to explore the roles of mitochondrial specific ERK1/2 activation on ischemic resistance.

Because it is well known that ERK1/2 is activated in DM cardiomyopathy [15], it is possible that mitochondrial ERK1/2 in DM hearts has already been facilitated to activate to promote ischemic resistance. Despite ERK1/2 being involved in the progression of DM cardiomyopathy, it has been noted that activation of ERK1/2 affords the pro-survival effects against ischemic injury as well [15]. We consider mitochondrial ERK1/2 phosphorylation may be responsible for ischemic resistance in DM hearts, because the ischemic stimulation increased int-renin (non-secreting and/or secreting) expression (Figure 1d) and r-renin can activate mitochondrial ERK1/2 (Figure 2e). However, we cannot deny the possibility of artificial bias in our results, because recombinant rat renin (r-renin), which we applied to isolated mitochondria, is a secreting form of renin, normally located in Golgi apparatus with glycosylation, and cannot enter mitochondria [1]. Further examination will be required to explore the effects of renin transcript codes for exon (1A-9), which is a non-secreting type of int-renin, on mitochondrial ERK1/2 activation during ischemia.

### 3.3. Int-Renin Inhibits Mitochondrial Permeability Transition Pore Opening through Mitochondrial ERK1/2 Activation

Opening mPTP during ischemia was evaluated as a final effector of cardiac injury. R-renin inhibited not only Atr-induced ΔΨ_m_ depolarization (Figure 3a) but also mitochondrial swelling (Figure 3b,c) in a U0126-sensitive manner, suggesting that int-renin inhibits mPTP opening through mitochondrial ERK1/2 activation. Because Atr is a ligand of adenine nucleotide translocator, which inhibits ADP/ATP translocation and in turn induces pore formation [16,17], Atr-induced mPTP opening may not reflect the ischemic cardiac injury in vivo. However, at least int-renin suppressed Atr-induced mPTP opening under our experimental conditions. Several lines of evidence support that mitochondrial ERK1/2 contributes to the inhibition of mPTP opening [10,18]. Although previous investigations suggested the contribution of survivor activating factor enhancement (SAFE) pathway on cardiac protection against ischemia/reperfusion such as ischemic preconditioning and pharmacological preconditioning [19], DM heart did not activate the signal transducer and activator of transcription 3 (STAT3) after ischemia in our experimental condition (Supplementary Materials Figure S5).

Compatible with a previous study [20], mitochondria from DM hearts exhibited mild ΔΨ_m_ depolarization at the baseline (Figure 2d and Figure 3a). There is one possibility that mild ΔΨ_m_ depolarization itself may be responsible for ischemic resistance in DM hearts. This is because mitochondrial Ca^2+^, which is one of the most critical triggers of mPTP opening, is dependent on its own ΔΨ_m_, and decreased mitochondrial Ca^2+^ by ΔΨ_m_ depolarization may reduce sensitivities of mPTP opening. However, at least in the Atr-induced mitochondrial swelling assay, the sensitivities of mPTP opening were not altered between non-DM and DM hearts. Although we studied Goto-Kakizaki rat hearts as the type II DM model, we assume that int-renin may have some contribution to protect heart from ischemia in type I model animals as well. Because previous investigation reported the cardiac ischemic resistance in type I DM model animals [13] and treating H9c2 myocytes with high glucose increased int-renin (Supplementary Materials Figure S3). Further investigations are required to explore precise molecular mechanisms by which int-renin suppresses mPTP opening, and to identify responsible factors by which int-renin inhibits mPTP opening in DM hearts.

## 4. Materials and Methods

### 4.1. Isolated Rat Heart Preparations and Measurement of Cardiac Performance

This investigation conformed to the National Institute of Health Guide for the Care and Use of Laboratory Animals [21] and was approved by the Hamamatsu University School of Medicine Animal Care and Use Committee (Approval code: 2017007; Approval date: 31/3/2017).

The isolation of heart and hemodynamic measurements in Langendorff-perfused hearts has been described previously [4]. For ischemic hearts, after 20 min of stabilization, the left anterior descending (LAD) coronary artery was occluded near its origin for 70 min. Direct renin inhibitor (DRI; aliskiren, 1 µM) was added 20 min before coronary ligation, and U0126 (an inhibitor of mitogen-activated protein kinases/extracellular signal-regulated kinase, 0.5 µM) was added before and after ligation.

### 4.2. Tissue from Ischemic Hearts and Mitochondrial Preparation

To evaluate intracellular signaling during ischemia, tissue from ischemic hearts was obtained. After 70 min of LAD ligation, the hearts were rapidly placed in ice-cold phosphate-buffered saline and then divided into the ischemic area and non-ischemic area. Isolated mitochondria from non-DM and DM hearts were prepared as described previously [4,22]. Briefly, heart tissue lysates were obtained from homogenized samples using the Mitochondria Isolation Kit for Tissue (Thermo Fisher Scientific Inc., Waltham, MA, USA). After a centrifugation at 700× *g* for 10 min, the supernatant was centrifuged at 3000× *g* for 15 min. Yielding pellet was the isolated mitochondria. Mitochondrial separation and integrity, which were ensured by western bolt analysis and electron microscope, respectively, were provided in Supplementary Materials Figure S2.

### 4.3. Western Blotting

Western blot analysis in whole cells and the mitochondrial fraction was performed as previously described [4,22]. Intracellular renin (int-renin) was assessed with primary antibody against renin, which was purchased from AnaSpec (Fremont, CA, USA). Primary antibodies against Phosphorylated-ERK1/2, ERK1/2, phosphorylated-Akt (Ser473), Akt, phosphorylated-p38, and p38 were purchased from Cell Signaling Technology, Inc. (Danvers, MA, USA). The antibody against actin was acquired from Santa Cruz Biotechnology (Dallas, TX, USA), prohibitin antibody was from Abcam (Cambridge, UK), and horseradish peroxidase-conjugated secondary antibodies were from Promega (Madison, WI, USA). Immunoblotting was performed using an enhanced Can Get Signal kit (TOYOBO, Osaka, Japan). Densitometric analysis was performed using the Molecular Imager ChemiDoc™ system (Bio-Rad Laboratories, Hercules, CA, USA).

### 4.4. Measurement of Mitochondrial Membrane Potential (ΔΨ_m_) and mPTP Opening

The mitochondrial membrane potential (ΔΨ_m_) in isolated mitochondria were measured with 5,5′,6,6′-tetrachloro-1,1′,3,3′-tetraethylbenzimidazol-carbocyanine iodide (JC-1), as described previously [4]. Isolated mitochondria were incubated with JC-1 (10 µM) for 30 min, and then rat recombinant renin (r-renin; 100 ng/mL) was applied according to the protocol. In some experiments, atractyloside (Atr; 50 µM, an opener of mPTP) and/or cyclosporine A (CysA; 1 µM, an inhibitor of mPTP) were applied to evaluate the mPTP opening. U0126 (1 µM) was added in some experiments to assess involvement of ERK1/2 activation. As a reference, an uncoupler, 2,4-dinitrophenol (DNP, 100 µmol/L) was applied to each group Fluorescent intensity was measured with a microplate reader (Synergy HT; BioTek Instruments, Inc., Winooski, VT, USA). JC-1 was excited at 480/528 nm, and emission signals were collected with 530/590 nm band-pass filters.

Mitochondrial permeability transition was also assessed by a mitochondrial swelling assay, and changes in absorbance at 540 nm were monitored for 30 min. Swelling assays were performed with an internal solution, containing (in mM): 50 KCl, 80 K-aspartate, 2 Na-pyruvate, 20 4-(2-hydroxyethyl)-1-piperazineethanesulfonic acid (HEPES), 3 MgCl_2_-6H_2_O, 2 Na2ATP, 3 EGTA, and 177 CaCl_2_ (pH 7.3) in the presence of other compounds (Atr, CysA, renin, and U0126) according to the study protocol.

### 4.5. Chemicals

U0126 was purchased from Cell Signaling Technology, JC-1 dyes from Thermo Fisher Scientific, Atr from Cayman (Ann Arbor, MI, USA), CysA from Sigma-Aldrich (St. Louis, MO, USA), DRI from Toronto Research Chemicals (Toronto, ON, Canada), and r-renin from AnaSpec (Fremont, CA, USA).

### 4.6. Data Analyses

Data are presented as the means ± standard error of the mean (SEM), and the number of cells or experiments is shown as n. Statistical analyses were performed using the *t*-test, one-way analysis of variance (ANOVA), followed by the Bonferroni’s test or two-way ANOVA according to the study protocol. Data are analyzed by Graphpad Prism, version 6.04 (Graphpad Software Inc., La Jolla, CA, USA). A significance level of *p* < 0.05 was considered statistically significant.

## 5. Conclusions

We conclude that int-renin accumulation in mitochondria during ischemia activates mitochondrial ERK1/2, hyperpolarizes ΔΨ_m_ and suppresses mPTP opening, which may be involved in the ischemic resistance in DM hearts.

## Figures and Tables

**Table 1:** Characteristics and laboratory data from non-diabetes mellitus (DM) and DM rats. In non-DM (*n* = 5) and DM rats (*n* = 5), heart weight (HW) and body weight (BW) were measured with blood samples before sacrifice. Data are shown as the means ± standard error of the mean (SEM). * *p* < 0.05 vs. non-DM by *t*-test.

	Non-DM	DM
Glucose (mg/dL)	254.0 ± 8.5	390.4 ± 15.9 *
Insulin (ng/mL)	1.72 ± 0.09	0.62 ± 0.08 *
Glycoalbumin (%)	1.30 ± 0.11	1.82 ± 0.14 *
Glycohemoglobin (%)	5.09 ± 0.02	5.65 ± 0.03 *
Body weight (g)	254.0 ± 2.4	278.0 ± 3.7 *
Heart weight (g)	0.89 ± 0.03	1.08 ± 0.03 *
HW/BW (×1000)	3.49 ± 0.11	3.87 ± 0.09 *

## Supplementary Materials

The following are available online at www.mdpi.com/1422-0067/19/1/55/s1.
