# Supplementary material for: Intracellular Renin Inhibits Mitochondrial Permeability Transition Pore via Activated Mitochondrial Extracellular Signal-Regulated Kinase (ERK) 1/2 during Ischemia in Diabetic Hearts
Source: Int J Mol Sci. 2017 Dec 25;19(1):55. doi: 10.3390/ijms19010055 (PMC5796005; doi:10.3390/ijms19010055)

Supplementary Materials:

Supplemental Figure S1

Baseline hemodynamic data in DM and non-DM heart

(a) Representative recordings of left ventricular (LV) pressure in Langendorff-perfused hearts from non-DM and DM rats.

(b, c) Time courses of changes in left ventricular developed pressure (b: LVDP) and left ventricular end-diastolic pressure (c: LVEDP) both in non-DM (●, n = 4) and DM hearts (○, n = 4). Values are the means ± SEM.

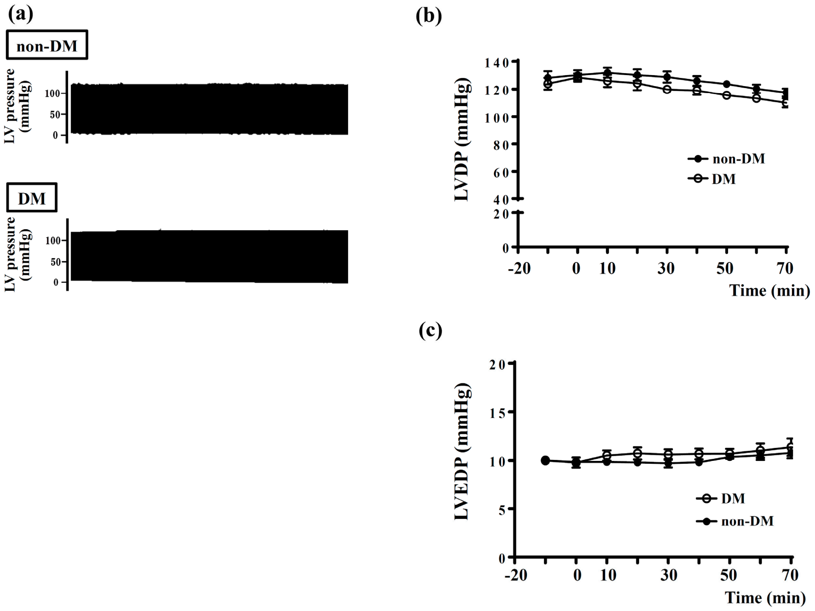

Supplemental Figure S2

Mitochondrial isolation and integrity in heart

(a) Representative western blot image of actin (cytosolic protein) and prohibitin (mitochondrial protein) in whole cell lysate, and cytosolic fraction, and mitochondrial fraction in non-DM hearts.

(b) Representative electron microscopic image (×20000) of isolated mitochondria from non-DM heart.

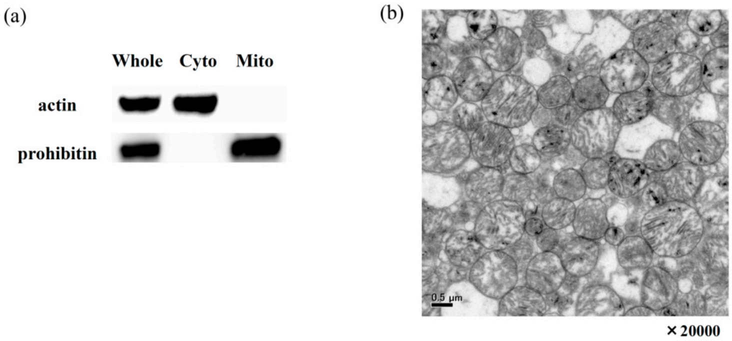

Supplemental Figure S3

High-glucose incubation increased int-renin and protected myocytes from hypoxia/reoxygenation

(a) Representative microscopic images of H9c2 myocytes with (lower) and without (upper) hypoxia/reoxygenation (H/R: 24 hr hypoxia via followed by 24 hr reoxygenation) both in normal- and high-glucose treated H9c2 myocytes. As reported previously, differentiated H9c2 myocytes were produced by treating H9c2 myoblasts with a differentiation medium for 5–7 days (Sakamoto et al., Exp Cell Res, 2015: 351, 109-120),

and then cells were incubated with high-glucose (25 mM) or normal-glucose (5 mM) medium for 24 hr. H/R was promoted using AnaeroPack® system (Mitsubishi gas chemical co. inc, Japan) according with the manufacturer's instruction.

(b-e) Western blot analysis in high- and normal-glucose treated H9c2 myocytes with and without H/R. Data represent the fold-increase of renin/actin (c), phosphorylated (P-ERK)/total ERK1/2 (T-ERK; d), and cleaved caspase-3/caspase-3 (e) from normal-glucose treated cells without H/R, and values are the means ± SEM of 4 independent experiments. *$p < 0.05$, †$p < 0.01$ vs. low-glucose treated cells without H/R (one-way ANOVA).

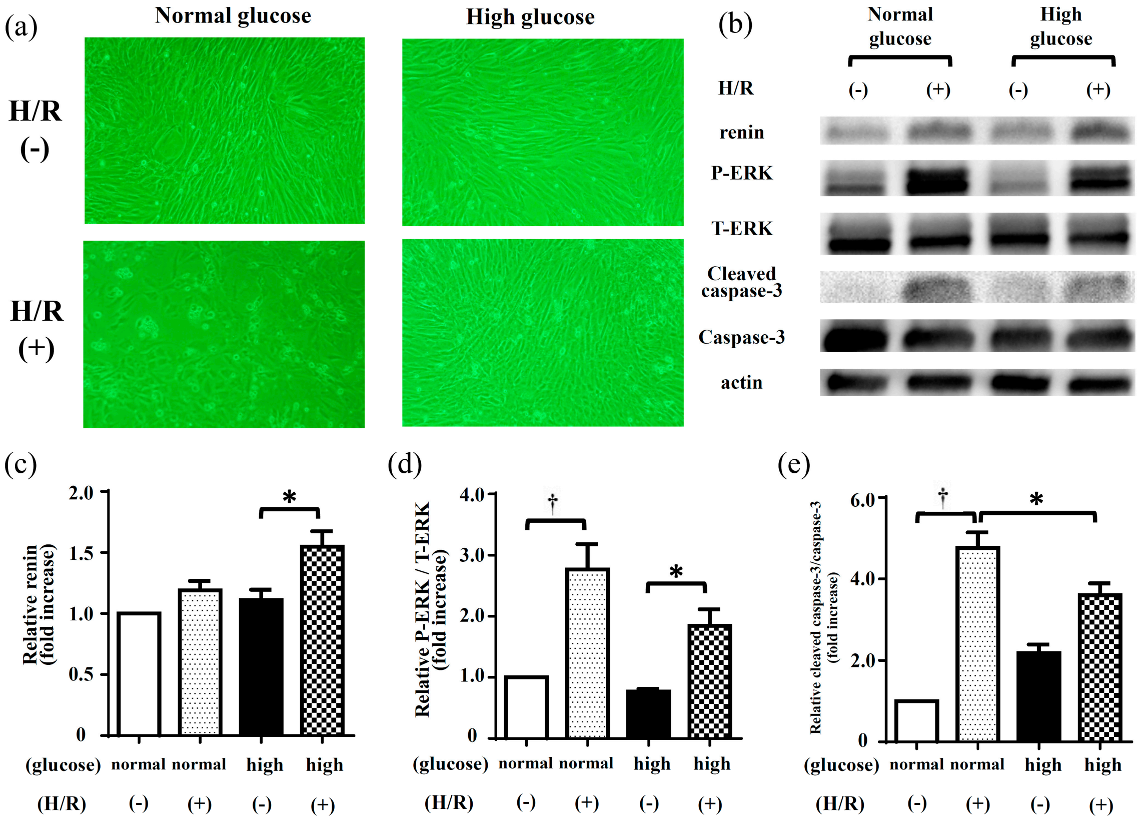

Supplemental Figure S4

Suppression of int-renin attenuated high-glucose-mediated myocytes protection

(a) Western blot analysis of int-renin in renin-siRNA expressing H9c2 myocytes. Data represent the fold-increase of renin/actin, and values are the means ± SEM of 4 independent experiments. *$p < 0.05$ (one-way ANOVA).

(b-d) Western blot analysis in renin-siRNA expressing H9c2 myocytes. The H9c2 myocytes expressing renin-siRNA or control-siRNA were treated with high-glucose, and then exposed to H/R. Data represent the fold-increase of phosphorylated/total ERK1/2 (c), and cleaved caspase-3/caspase-3 (d) from control-siRNA expressing cells without H/R, and values are the means ± SEM of 4 independent experiments. *$p < 0.05$, †$p < 0.01$ (one-way ANOVA).

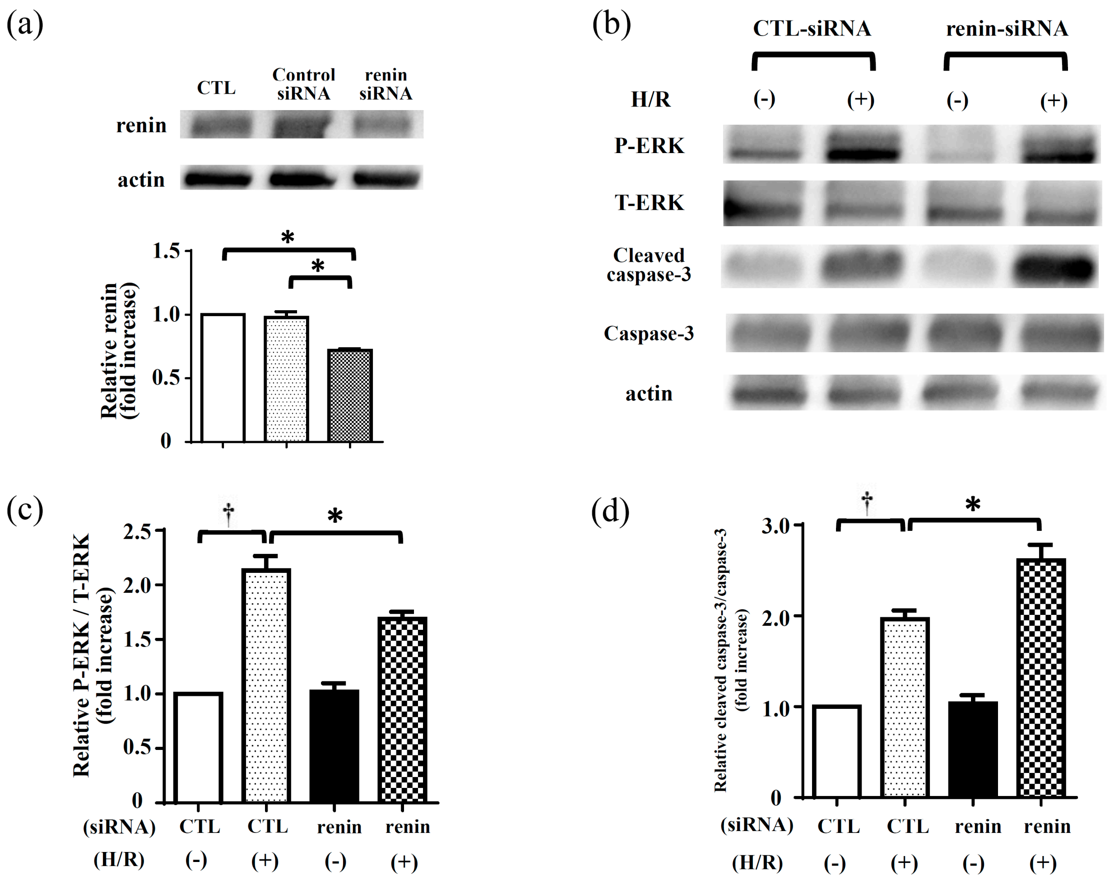

Supplemental Figure S5

Signal transducer and activator of transcription-3 (STAT3) activation in DM heart after ischemia. Western blot analysis of STAT-3 activation in whole cell lysate (a) and mitochondrial fraction (b) in both non-DM and DM hearts. Hearts were subjected to ischemia for 70 min, and phosphorylation levels of STAT3 (serine 727) were assessed among the control, non-ischemic area (non-is), and ischemic area (is). Data represent the fold-increase of phosphorylated (: P-STAT3)/total STAT3 (T-STAT3) from non-ischemic controls, and values are the means ± SEM from 3 independent experiments.

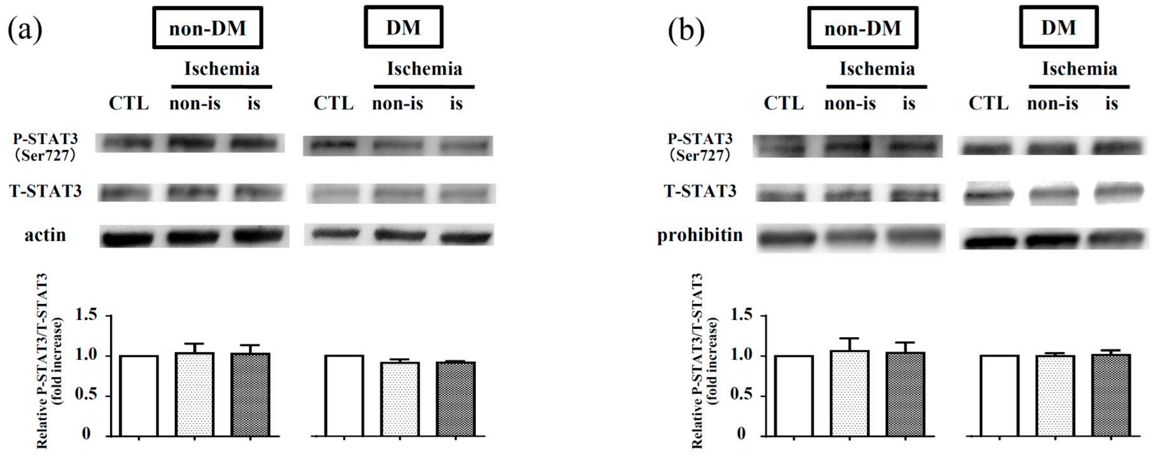

Supplement: Supplementary file 1 [file ijms-19-00055-s001.pdf]
